# Efficient targeted mutagenesis in allotetraploid sweet basil by CRISPR/Cas9

**DOI:** 10.1002/pld3.233

**Published:** 2020-06-11

**Authors:** Natasha Navet, Miaoying Tian

**Affiliations:** ^1^ Department of Plant and Environmental Protection Sciences University of Hawaii at Manoa Honolulu HI USA

**Keywords:** CRISPR/Cas9, gene editing, *ObDMR1*, *Ocimum basilicum*, sweet basil, transgene free

## Abstract

Sweet basil (*Ocimum basilicum*) is an economically important herb and its global production is threatened by basil downy mildew caused by the obligate biotrophic oomycete *Peronospora belbahrii*. Effective tools are required for functional understanding of its genes involved in synthesis of valuable secondary metabolites in essential oil and disease resistance, and breeding for varieties with improved traits. Clustered regularly interspaced short palindromic repeat (CRISPR)/Cas9 gene editing technology has revolutionized crop breeding and functional genomics. The applicability and efficacy of this genomic tool in the allotetraploid sweet basil were tested by editing a potential susceptibility (*S*) gene *ObDMR1*, the basil homolog of *Arabidopsis DMR1* (*Downy Mildew Resistant 1*) whose mutations conferred nearly complete resistance against *Arabidopsis* downy mildew pathogen, *Hyaloperonospora arabidopsidis*. Two single guide RNAs targeting two different sites of the *ObDMR1* coding sequence were designed. A total of 56 transgenic lines were obtained via *Agrobacterium*‐mediated stable transformation. Mutational analysis of 54 T0 transgenic lines identified 92.6% lines carrying mutations at target 1 site, while a very low mutation frequency was detected at target 2 site. Deep sequencing of six T0 lines revealed various mutations at target 1 site, with a complete knockout of all alleles in one line. *ObDMR1* homozygous mutant plants with some being transgene free were identified from T1 segregating populations. T2 homozygous mutant plants with 1‐bp frameshift mutations exhibited a dwarf phenotype at young seedling stage. In summary, this study established a highly efficient CRISPR/Cas9‐mediated gene editing system for targeted mutagenesis in sweet basil. This system has the capacity to generate complete knockout mutants in this allotetraploid species at the first generation of transgenic plants and transgene‐free homozygous mutants in the second generation. The establishment of this system is expected to accelerate basil functional genomics and breeding.

## 1 INTRODUCTION

Basil belonging to the genus *Ocimum* in *Lamiaceae* family is a popular herb widely used in culinary, therapeutic, and cosmetic industries (da Costa et al., 2015; Wyenandt et al., 2015). There are nearly 35–150 species of *Ocimum* distributed across the globe, among which *Ocimum basilicum,* commonly known as sweet basil, is highly demanded with significant economic value (Wyenandt et al., 2015). Essential oil extracted from sweet basil contains diverse secondary metabolites with a wide spectrum of biological activities, such as high antioxidant, antimicrobial, anticancer, and insecticidal activities, and thus used for culinary flavoring, aromatherapy, preservation of various foods, and eco‐friendly insect control (Li & Chang, 2016). Different ploidies have been reported across varied species of *Ocimum,* with *O. basilicum* considered tetraploid (2n = 4× = 48) (Pyne, Honig, Vaiciunas, Wyenandt, & Simon, 2018).

Global production of sweet basil is severely threatened by an obligate biotrophic oomycete pathogen *Peronospora belbahrii,* which causes basil downy mildew (BDM) (Cohen, Ben Naim, Falach, & Rubin, 2017; Wyenandt et al., 2015). Current control strategies for BDM mainly rely on the use of limited available fungicides, whose repeated use poses the risk of evolving fungicide‐resistant strains (Cohen et al., 2017; Wyenandt et al., 2015). In addition, frequent applications of these chemicals increase production cost, and adversely affect human health and the environment. The most potent and cost‐effective control strategy to halt the rapid dissemination of BDM is to utilize disease‐resistant cultivars. Resistance and tolerance have been found in *Ocimum* species differing vastly from sweet basil (Cohen et al., 2017). Traditional breeding involving interspecific hybridization to transfer naturally existing disease resistance genes to sweet basil has been a hassle as it is largely met with sexual incompatibility, hybrid F1 sterility, and difficulty in segregating out undesirable traits (Ben‐Naim, Falach, & Cohen, 2018; Cohen et al., 2017). Genetic engineering, with the advent of cutting‐edge clustered regularly interspaced short palindromic repeat (CRISPR)/CRISPR‐associated protein (Cas) gene editing technology, offers a promising platform to functionally understand the molecular basis of basil‐*P. belbahrii* interactions, broaden resistance resources, and accelerate the breeding process.

Adapted from bacterial immune systems, CRISPR/Cas has revolutionized the way scientists perform functional genomics studies and crop breeding as this technology provides an unparalleled tool to precisely edit DNA sequences with ease, high efficiency, and high fidelity (Zhou, Wang, & Liu, 2018). The first used CRISPR system for genome editing was CRISPR/Cas9 (Jinek et al., 2012, 2013), which has been successfully used in genome editing of many plant species (Haque et al., 2018; Jaganathan, Ramasamy, Sellamuthu, Jayabalan, & Venkataraman, 2018). This gene editing system requires Cas9 and a single guide RNA (sgRNA), which is a fusion of CRISPR RNA (crRNA) containing a 20‐nt DNA target sequence upstream of a Cas9 protospacer adjacent motif (PAM, 5′‐NGG‐3′) and trans‐activating CRISPR RNA (tracrRNA) (Jinek et al., 2012). This technology relies on specific base pairing of the 20‐bp sequence of the sgRNA with the target DNA, which directs Cas9 endonuclease to cleave the target DNA at 3‐nt upstream of the PAM motif (Jiang & Doudna, 2017). The double‐stranded breaks (DSBs) generated by Cas9 activate innate DNA repair by either non‐homologous end‐joining (NHEJ) or homology‐directed repair (HDR) mechanism (Jiang & Doudna, 2017). Without a homologous DNA template, the cell repairs the DSB through NHEJ, which is error‐prone causing short insertions or deletions (indels) around the cleavage site. With a homologous DNA template, the cell will repair the DSB through HDR, leading to precise mutations. As this approach can generate homozygous or complete knockout mutants as early as in the first generation of transgenic lines for both diploid and polyploid species (Gumtow, Wu, Uchida, & Tian, 2018; Pan et al., 2016; Wang et al., 2016), it greatly speeds up functional genomics studies and shortens the breeding process. Furthermore, the use of CRISPR/Cas9‐mediated gene editing allows for the development of foreign DNA‐free crops, which is more acceptable by consumers, as opposed to the conventional way of developing genetically modified (GM) crops. Traditional genetic engineering generates GM crops that embody the transgene within the genome to express new traits. In contrast, the trait generated through CRISPR‐mediated gene editing can be segregated from the introduced transgenes; or the desired trait can be achieved via DNA‐free approach for delivery of gene‐editing reagents. The resultant transgene‐free plants can bypass the regulatory restrictions set for GM crops by USDA (Waltz, 2016). Many proof‐of‐concept studies have used CRISPR for crop nutritional improvement and enhanced resistance to biotic and abiotic stresses (Borrelli, Brambilla, Rogowsky, Marocco, & Lanubile, 2018; Jaganathan et al., 2018; Langner, Kamoun, & Belhaj, 2018).

Many pathogens exploit plant genes for successful infection and colonization. Plant genes that facilitate pathogen infection are considered as plant susceptibility (S) genes (van Schie & Takken, 2014). The modification or removal of *S* genes prevents pathogen infection, and therefore represents an effective strategy to achieve resistance (Dong & Ronald, 2019; Zaidi, Mukhtar, & Mansoor, 2018). Targeted mutagenesis of *S* genes using CRISPR/Cas technology has recently emerged as a desirable approach to generating broad‐spectrum disease resistance (Borrelli et al., 2018; Zaidi et al., 2018). The success of this approach relies on the targeting of a suitable *S* gene. Due to the lack of an effective functional genomics approach for sweet basil, a *S* gene that contributes to susceptibility to BDM has not been identified. However, a number of *S* genes have been identified in *Arabidopsis* that are required for susceptibility to its downy mildew‐causing oomycete pathogen *Hyaloperonospora arabidopsidis,* one of which is *DMR1* (*Downy Mildew Resistant 1*) (van Damme, Huibers, Elberse, & Van den Ackerveken, 2008; van Damme et al., 2009; Hok et al., 2011; Van Damme et al., 2005). *DMR1* encodes a homoserine kinase (HSK) catalyzing phosphorylation of homoserine. *dmr1* mutants contain high levels of homoserine and are highly resistant to *H. arabidopsidis* (Van Damme et al., 2005, 2009). *DMR1* seems to be conserved in various plant species and its homologs in multiple plant species have been shown to be a determining factor for susceptibility to various pathogens (Huibers et al., 2013; Sun et al., 2016). Therefore, the *DMR1* homolog in sweet basil, *ObDMR1*, represents a candidate *S* gene with a potential role in susceptibility to BDM.

In the present study, we targeted *ObDMR1* to establish an effective CRISPR/Cas9‐mediated gene editing system in sweet basil. Sweet basil cultivar Genoveser was transformed, respectively, with two sgRNA/Cas9 binary constructs targeting one or two sites of *ObDMR1* via *Agrobacterium*‐mediated stable transformation. High frequency of small insertion/deletion (indel) mutations and complete knockout of *ObDMR1* were achieved in the first generation (T0) of transgenic plants. Homozygous *ObDMR1* mutants with some being transgene free were identified in the second generation (T1). T2 homozygous mutants exhibited a dwarf phenotype at the young seedling stage, confirming the efficient mutagenesis of *ObDMR1* by CRISPR/Cas9.

## 2 MATERIALS AND METHODS

### 2.1 Plant materials and growth conditions

Sweet basil cultivar Genoveser (Enza Zaden) plants were used for *Agrobacterium*‐mediated transformation, and routinely grown in a controlled growth chamber set at 25°C with a photoperiod of 12 hr. The same conditions were applied to grow newly regenerated transgenic T0, T1, and T2 seedlings. Older T0 and T1 plants were grown in the greenhouse at 25–27°C with a photoperiod of 16 hr for seed production. Selfing bags were mounted on flower stalks at the beginning of flowering to avoid cross‐pollination.

### 2.2 Identification of the homolog of Arabidopsis DMR1 in sweet basil

The homolog of *Arabidopsis DMR1* (*AtDMR1*) in sweet basil (*Ocimum basilicum*), *ObDMR1,* was identified by TBLASTX search against the non‐redundant transcriptomic sequence dataset generated from two sweet basil varieties Red Rubin and Tigullio using Trinity assembly (Torre et al., 2016) using the protein encoding sequence of *AtDMR1* (GenBank accession: NM_127281) as a query. A single significant hit was identified and the transcript sequence was retrieved. To amplify *ObDMR1* from sweet basil cv. Genoveser by PCR, we designed a pair of primers (*DMR1*‐Gen‐F: 5′‐CGTCCCCTATTCTCTCACTATGGC‐3′; *DMR1*‐Gen‐R: 5′‐AAAACCCAGAGACCATGCAAATG‐3′) targeting 5′‐UTR and 3′‐UTR of this transcript. PCR was performed using Genoveser genomic DNA (gDNA) as the template and Phusion High‐Fidelity DNA Polymerase (NEB) with PCR conditions as: initial denaturation at 94°C for 3 min; followed by 35 cycles at 94°C for 15 s, 54°C for 30 s and 72°C for 75 s; with a final extension at 72°C for 7 min. The resultant PCR product was gel purified using QIAquick Gel Extraction Kit (QIAGEN) and subjected to Sanger sequencing. The amino acid sequences of ObDMR1 in Genoveser and AtDMR1 were aligned using BLASTP to assess their homology. The amino acid sequence alignment was also generated using CLUSTALX 2.1 (Larkin et al., 2007) and displayed using BOXSHADE (https://embnet.vital‐it.ch/software/BOX_form.html).

### 2.3 Selection of sgRNA target sequences for editing ObDMR1 using CRISPR/Cas9

Single guide RNA target sites were identified by searching 20‐nt sequences immediately upstream of the PAM sequence 5′‐NGG‐3′ within the coding region of Genoveser *ObDMR1* using Eukaryotic Pathogen CRISPR guide RNA design tool (EuPaGDT) (http://grna.ctegd.uga.edu/) (Peng & Tarleton, 2015) with default parameters. The non‐redundant transcriptomic assembly of two sweet basil varieties Red Rubin and Tigullio generated using Trinity (Torre et al., 2016) was uploaded as a custom genome to identify potential off‐targets. The candidate target sequences with total score as well as efficiency score of more than 0.50 were selected and further subjected to secondary structure analyses using the web tool RNAstructure (Reuter & Mathews, 2010) (http://rna.urmc.rochester.edu/RNAstructureWeb/Servers/Predict1/Predict1.html). The ones with no more than three hydrogen bonds were chosen for sgRNAs.

### 2.4 Vector construction

The constructs for CRISPR/Cas9‐mediated gene editing of *ObDMR1* were generated using plant binary vector pKSE401 as described by Xing et al. (2014). For generating the construct expressing one sgRNA (sgRNA1), we used the oligo pair *DMR1*‐target1‐*F* (5′‐ATTGTTTCCATTTCCAACATCAC‐3′) and *DMR1*‐target1‐R (5′‐AAACGTGATGTTGGAAATGGAAA‐3′). The underlined sequence represents the target site of sgRNA1. The oligos were annealed to produce a double‐stranded fragment with 4‐nt 5′ overhangs at both ends and then ligated into the BsaI‐digested pKSE401. The resulting plasmid was named as pKSE401‐sgRNA1.

For generating the construct expressing two sgRNAs (sgRNA1 and sgRNA2), four oligos (DT1_BsF: 5′‐ATATATGGTCTCGATT**GTTTCCATTTCCAACATCAC**GTT‐3′; DT1‐F0: 5′‐T**GATTGTTTCCATTTCCAACATCAC**GTTTTAGAGCTAGAAATAGC‐3′; DT2‐R0: 5′‐AACTTTGGAATTGCGCCGGCATCAATCTCTTAGTCGACTCTAC‐3′ and DT2_BsR: 5′‐ATTATTGGTCTCTAAACTTTGGAATTGCGCCGGCATCAA‐3′) were designed as described by Xing et al. (2014). The bold underlined and underlined represent the target sequences of sgRNA1 and sgRNA2, respectively. The DNA fragment containing sgRNA1, U6‐26 terminator, U6‐29 promoter and the target sequence of sgRNA2 was amplified using PCR with these four oligos and pCBC‐DT1T2 (Xing et al., 2014) as a template. The PCR reaction was performed using Phusion High‐Fidelity DNA Polymerase (NEB) with cycling conditions as: 98°C for 2 min; followed by 35 cycles at 98°C for 30 s, 71°C for 15 s and 72°C for 90 s; with a final extension at 72°C for 7 min. The PCR product was purified using QIAquick PCR Purification Kit (QIAGEN) and then digested by BsaI. The digested PCR product was ligated into BsaI‐digested pKSE401 vector. The resulting plasmid was named as pKSE401‐sgRNA1+2. Both pKSE401‐sgRNA1 and pKSE401‐sgRNA1+2 were introduced into *Agrobacterium tumefaciens* strain EHA105 for basil transformation.

### 2.5 Agrobacterium‐mediated transformation of sweet basil

Sweet basil cultivar Genoveser was transformed with *A. tumefaciens* EHA105 harboring pKSE401‐sgRNA1 or pKSE401‐sgRNA1+2 based on the method described previously (Deschamps & Simon, 2002; Phippen & Simon, 2000) with modifications. The *Agrobacteria*, stored at −80°C, were streaked on LB plate supplemented with 50 μg/ml kanamycin and 15 μg/ml rifampicin and grown for 2 days at 28°C. A day prior to basil transformation, colonies were spread to a new plate and incubated at 28°C overnight. On the day of basil transformation, bacterial cells were scraped from the overnight plate and suspended in *Agrobacterium* inoculation media (IN) (4.3 g/L Murashige and Skoog basal medium [MS], 3% sucrose, 16.8 μM thidiazuron [TDZ], and pH 5.7, supplemented with 200 μM acetosyringone) to OD_600_ of 0.6. This suspension was incubated at room temperature (RT) in dark with gentle shaking at 70 rpm for 2 hr. Meanwhile, the first pair of true leaves from 3‐week‐old Genoveser plants were plucked and surface sterilized in 12% (v/v) Clorox solution for 5 min. Two explants were excised from regions close to leaf base along the midrib of each leaf using cork borer number 2. After the completion of incubation of *Agrobacterium* suspension, the explants were immersed in this suspension for 30 min at RT. Explants were then taken out and excess suspension was removed by pressing the explants gently between two layers of sterile filter paper. These explants were co‐cultivated with *Agrobacteria* on callus and shoot induction (SI) media (4.3 g/L MS, 3% sucrose, 0.8% agar, 16.8 μM TDZ, pH 5.7) supplemented with 200 μM acetosyringone, with the abaxial side facing the media, for 3 days in dark at 25°C. For induction and selection of transgenic calli and shoots, explants were transferred to SI media supplemented with 200 μg/ml timentin and 30 μg/ml kanamycin), and grown for 4–6 weeks in dark at 25°C with sub‐culturing every 2 weeks onto fresh media. Once the shoots developed on calli, they were transferred to root induction (RI) media (4.3 g/L MS, 3% sucrose, 0.8% agar, 0.054 μM NAA [1‐napthaleneacetic acid], pH 5.7) supplemented with 200 μg/ml timentin and 30 μg/ml kanamycin, and grown for 1 week in dark at 25°C, and later under a 12‐hr photoperiod cycle for 4–8 weeks with regular sub‐culturing performed every 2 weeks. Plantlets with properly defined root and shoot systems were removed from media jars, washed thoroughly with water to remove the media, and then planted in moistened soil (SunGro Horticultutre Sunshine Mix #4). The plants were grown under 100% relative humidity for 3–4 days in a tray covered with a plastic dome in a growth chamber set at 25°C with a 12‐hr photoperiod. Humidity was gradually reduced over next 2–3 days and then the plants were transferred to the greenhouse to produce seeds.

### 2.6 Detection of transgene integration and ObDMR1 mutations in transgenic plants

DNA isolation was performed using approximately 50 mg leaf tissue of regenerated basil plants (T0 and T1), which was smashed with a stainless steel ball (5 mm) in 400 μl of DNA extraction buffer (200 mM Tris–HCl, pH 7.5, 250 mM NaCl, 25 mM EDTA, and 0.5% SDS) using FastPrep‐24 (MP Biomedicals) at 4.0 m/s for 20 s. The following DNA isolation procedures were performed as described previously (Shao & Tian, 2018). This crude gDNA was used as the template to determine the transgene integration in T0 and T1 plants by PCR. PCR was performed using primers U6‐26p‐*F* (5′‐TGTCCCAGGATTAGAATGATTAGGC‐3′) (Xing et al., 2014) & *DMR1*‐target1‐R for plants transformed with pKSE401‐sgRNA1, and primers U6‐26p‐F and U6‐29p‐R (5′‐AGCCCTCTTCTTTCGATCCATCAAC‐3′) (Xing et al., 2014) for plants transformed with pKSE401‐sgRNA1+2. To identify the mutations in *ObDMR1*, the primers *DMR1*‐CRISPR‐*F* (5′‐CCCGTCTTCTCCTCCGTCAAATC‐3′) and *DMR1*‐CRISPR‐R (5′‐AGTTCTGACGGCGACAGAGGACC‐3′) flanking both sgRNA1 and sgRNA2 target sites (Figure 2a) were used for PCR to amplify the *ObDMR1* fragment. PCR was performed using Phusion high‐fidelity DNA polymerase (NEB). The PCR products were treated with ExoSAP‐IT (ThermoFisher Scientific) followed by Sanger sequencing using the above primers. The Synthego ICE v1.1 CRISPR Analysis Tool (https://ice.synthego.com/) (Hsiau et al., 2018) was used for chromatogram decoding to assess indel frequency. Sequence trace files from the transgenic lines and wild‐type (WT) were uploaded to the server for the analysis.

PCR products from six T0 transgenic lines with highest indel frequency were purified using QIAGEN Gel extraction kit and subjected to amplicon deep sequencing for detailed mutation analyses. DNA library preparations, sequencing reactions, and adapter sequence trimming were conducted at GENEWIZ, Inc. DNA library preparation was performed using NEBNext Ultra DNA Library Prep kit following the manufacturer's recommendations (Illumina). Briefly, end repaired adapters were ligated after adenylation of the 3′ends followed by enrichment by limited cycle PCR. DNA libraries were validated and quantified before loading. The pooled DNA libraries were loaded on the Illumina instrument according to manufacturer's instructions. The samples were sequenced using a 2 × 250 bp paired‐end (PE) configuration. Image analysis and base calling were conducted by the Illumina Control Software on the Illumina instrument. Approximately 50,000 amplicon reads was obtained after trimming for each independent line. Unique sequences with more than 0.5% of total reads were analyzed in detail to determine the types of mutations at the targeted sites of *ObDMR1*.

### 2.7 Morphological phenotyping of T2 homozygous mutant plants

T2 seeds of T1 homozygous mutant lines along with WT were sown at the same time under the same growth conditions set at 25°C with a photoperiod of 12 hr to monitor the germination rate. Growth and development phenotypes were monitored from 2 weeks until 10 weeks. The height of 18‐day‐old plants was measured from the base to the tip of the stem. A representative plant from each individual T1 homozygous mutant line and WT was photographed after removing from the soil. The height measurements were subjected to one‐tailed *t* test analysis using SAS software to determine the statistical difference between the mutants and WT.

### 2.8 Accession numbers

The *ObDMR1* gDNA sequence was deposited in NCBI GenBank under accession number MT000722.

## 3 RESULTS

### 3.1 Identification of the homolog of Arabidopsis DMR1 in sweet basil

To determine whether a homolog of *Arabidopsis DMR1* (*AtDMR1*, At2g17265) is present in sweet basil, we did a local TBLASTX search using the 1113‐bp protein coding sequence of *AtDMR1* as a query against the non‐redundant transcriptomic sequence dataset generated from two sweet basil varieties Red Rubin and Tigullio using Trinity assembly (Torre et al., 2016). We identified one significant hit with an E value of 9e‐147. All other hits were insignificant with E values higher than 0.11. The single significant hit comp43301_c0_seq2 was a transcript of 1,616 bp, with a predicted open reading frame (ORF) of 1,137 bp. BLASTP search with its translated amino acid sequence as the query against NCBI non‐redundant database found that it was highly similar to HSK or HSK‐like proteins in various plant species. BLASTP against Araport11 protein sequences in The *Arabidopsis* Information Resource (TAIR) identified AtDMR1 as the best hit. These results suggested the identification of the homolog of *AtDMR1* in sweet basil, designated as *ObDMR1*.

**FIGURE 1:**
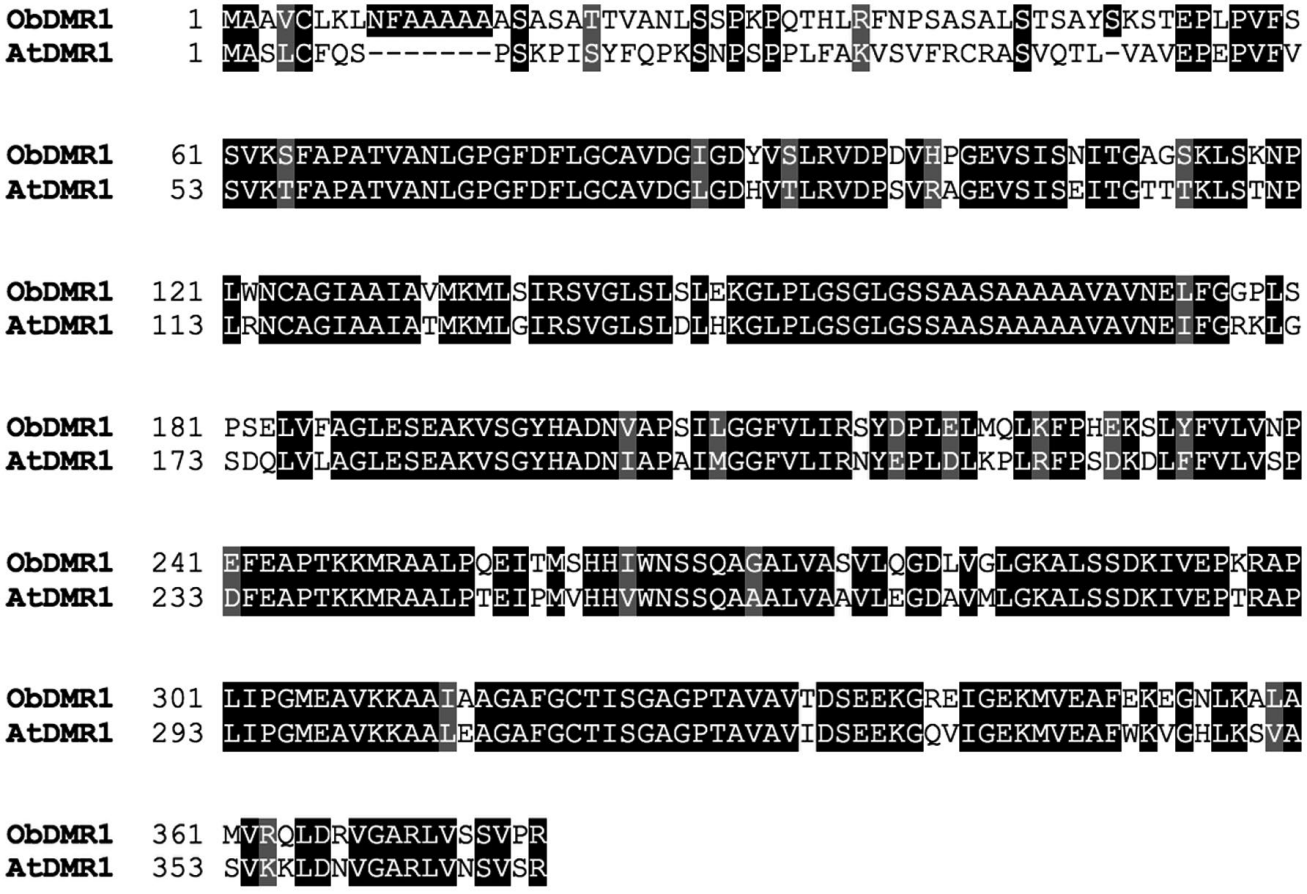
Amino acid sequence alignment of ObDMR1 and AtDMR1. Sequences were aligned using CLUSTALX 2.1 and displayed with BOXSHADE. The identical and similar amino acids are shaded

As we planned to perform CRISPR/Cas9‐mediated gene editing of *ObDMR1* in sweet basil cultivar Genoveser, we amplified and sequenced the *ObDMR1* gDNA using a pair of primers targeting the 5′‐UTR and 3′‐UTR of the *ObDMR1* transcript from Red Rubin and Tigullio. Genoveser *ObDMR1* gDNA was predicted to have an ORF of 1137‐bp encoding 361 amino acids. This predicted ORF aligned with the ORF of the *ObDMR1* transcript from Red Rubin and Tigullio with no gaps (Figure S1), suggesting that there is no intron present from translation start codon to stop codon. There were a total of 10 single nucleotide polymorphisms (SNPs) with only one leading to the difference in the third amino acid from N‐terminus, with alanine (A) in Genoverser and threonine (T) in Red Rubin and Tigullio (Figure S1). The amino acid sequence of ObDMR1 from Genoveser shares 78.59% identity with AtDMR1 with an E value of 2e‐168 (Figure 1). Similar to AtDMR1, a HSK domain belonging to PLN02451 superfamily was identified using NCBI Conserved Domain Search (Marchler‐Bauer et al., 2017).

### 3.2 Selection of sgRNA target sequences and generation of constructs for Agrobacterium‐mediated transformation

**FIGURE 2:**
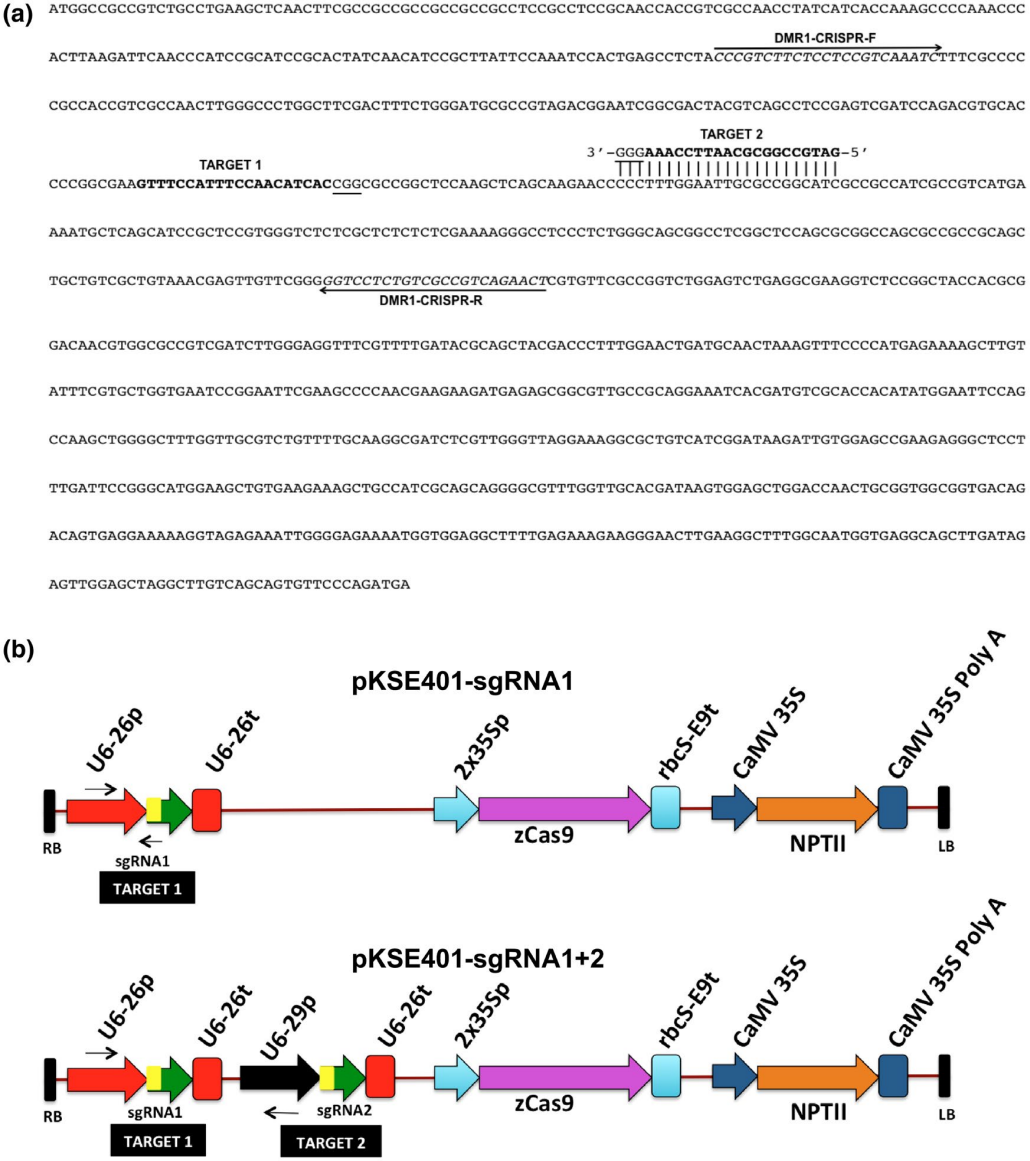
sgRNA target sites and constructs used for targeted mutagenesis of *ObDMR1*. (a) The protein encoding sequence of Genoveser *ObDMR1* with two sgRNA target sequences (target 1 and target 2) marked in bold and PAM sites underlined. The sequences of the primers (*DMR1*‐CRISPR‐F and *DMR1*‐CRISPR‐R) used to amplify the 383‐bp *ObDMR1* fragment for mutation analyses are shown with black arrows. (b) Schematic representations of expression cassettes within the T‐DNA of pKSE401‐sgRNA1 and pKSE401‐sgRNA1+2. The elements were described in Xing et al. (2014). The primer pairs (U6‐26p‐F and *DMR1*‐target1‐R, U6‐26p‐F and U6‐29p‐R) used for detecting the transgene integration in plants transformed with pKSE401‐sgRNA1 and pKSE401‐sgRNA1+2 are indicated by arrows. sgRNA, single guide RNA

To test the efficiency of CRISPR/Cas9‐mediated gene editing in sweet basil, *ObDMR1* was targeted for mutagenesis. 20‐nt candidate sgRNA target sequences were identified using the online tool EuPaGDT (http://grna.ctegd.uga.edu/) (Peng & Tarleton, 2015) and their RNA secondary structures were analyzed using RNAstructure (http://rna.urmc.rochester.edu/RNAstructureWeb/Servers/Predict1/Predict1.html) (Reuter & Mathews, 2010). As the whole genome sequence of sweet basil was not available, we used the transcriptomic sequence dataset of two sweet basil varieties Red Rubin and Tigullio (Torre et al., 2016) as the custom genome for off‐target analysis. We selected two target sequences for gene editing based on combined consideration of their locations, total scores, efficiency scores, GC content, absence of off‐targets and the number of hydrogen bonds in the predicted RNA structure. Both selected targets lie in regions encoding the N‐terminal half of the protein. Target 1 is located at 310–329 bp downstream of the translation start site on the sense strand, and target 2 is located at 362–381 bp downstream of the translation start on the complementary strand (Figure 2a). Both had total score and efficiency score higher than 0.5, and no off‐targets were found in the transcriptome of Red Rubin and Tigullio (Table S1). The GC content is 40% and 55% for target 1 and target 2, respectively. The predicted RNA structures of both targets were relatively less complex with three hydrogen bonds (Table S1).

To perform sweet basil gene editing using *Agrobacterium*‐mediated transformation, we utilized pKSE401, which is a plant binary vector developed for gene editing in dicots (Xing et al., 2014), to generate two constructs pKSE401‐sgRNA1 and pKSE401‐sgRNA1+2 that express one sgRNA and two sgRNAs, respectively. pKSE401‐sgRNA1 expresses sgRNA1 under the control of *Arabidopsis* U6‐26 promoter and U6‐26 terminator (Figure 2b). pKSE401‐sgRNA1+2 expresses two sgRNAs, with sgRNA1 under the control of *Arabidopsis* U6‐26 promoter and U6‐26 terminator, and sgRNA2 under the control of *Arabidopsis* U6‐29 promoter and U6‐26 terminator (Figure 2b). Both constructs express maize‐codon optimized Cas9 under the control of the double CaMV 35S promoter, and *NPTII* gene under the control of CaMV 35S promoter for selection of transgenic plants (Figure 2b). Both constructs were introduced into *Agrobacterium tumefaciens* strain EHA105, respectively, for basil transformation.

### 3.3 Generation of transgenic sweet basil plants expressing gene‐editing reagents

**FIGURE 3:**
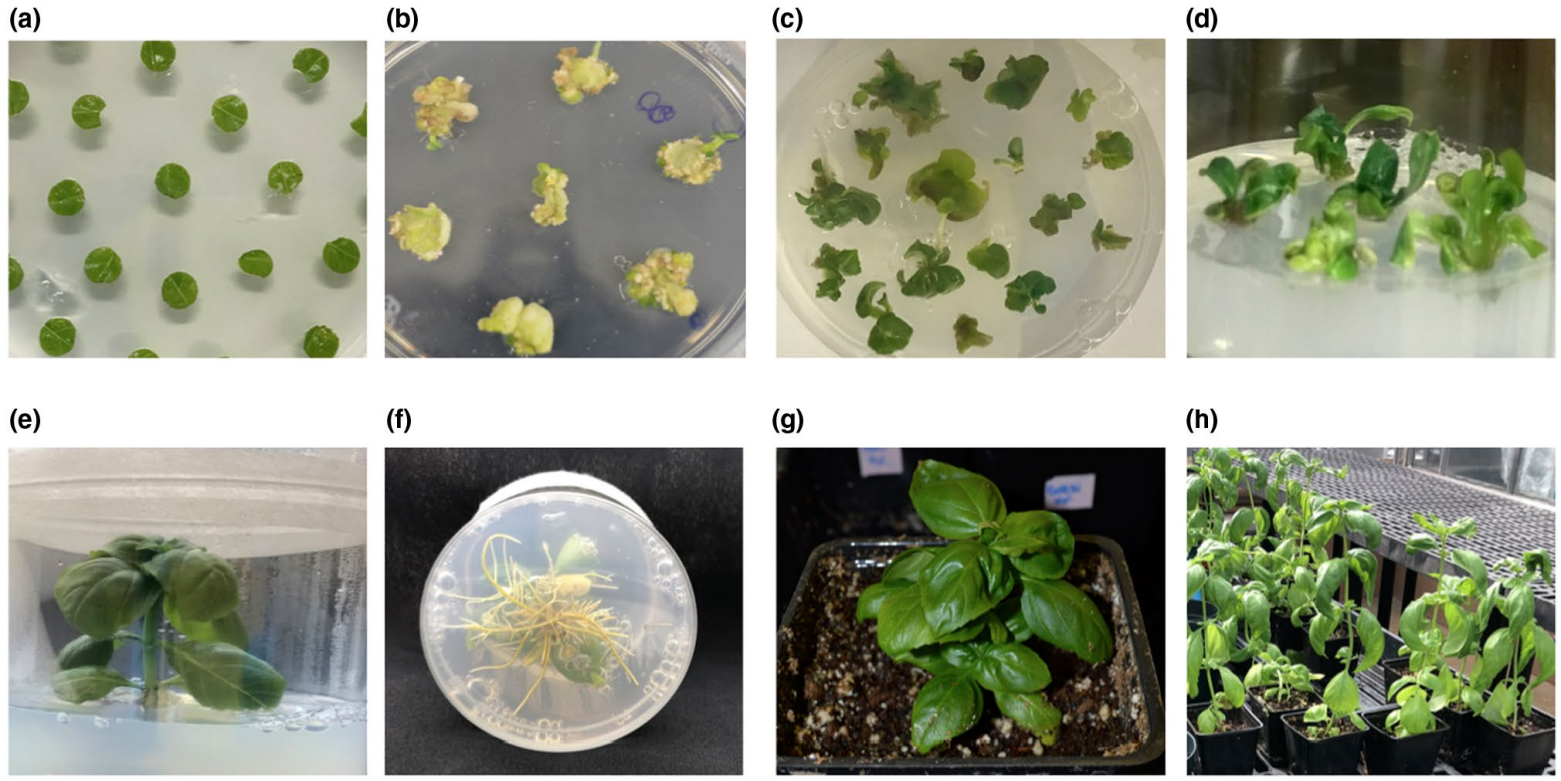
Transformation and regeneration of sweet basil. (a) Co‐cultivation of leaf explants with *Agrobacteria* on callus and shoot induction (SI) media containing acetosyringone in dark for 3 days. (b) Callus formation and shoot regeneration on SI media with kanamycin selection after 2 weeks in dark. (c) Individual sprouting shoots placed on root induction (RI) media with kanamycin for shoot elongation and root development. (d) Plantlets formed on RI media with kanamycin. (e) and (f) Well‐developed transgenic plants. (g) Acclimatization of a plantlet in soil. (h) Plants in the greenhouse for seed production

To generate transgenic basil plants expressing sgRNA(s) and Cas9, leaf discs prepared from the first pair of true leaves from 3‐week‐old Genoveser plants were used as explants. The explants were infected and co‐cultivated with *A. tumefaciens* EHA105 harboring either pKSE401‐sgRNA1 or pKSE401‐sgRNA1+2 on callus and shoot induction (SI) media supplemented with acetosyringone for 3 days (Figure 3a). Then the explants were transferred to SI media containing kanamycin to selectively induce the formation of transgenic calli and shoots. Calli were seen 2 weeks after culturing the explants on kanamycin‐containing SI media and tiny shoot buds emerged from calli later after subculturing on fresh media (Figure 3b). Shoots with emerging stalks were placed on root induction media (RI) (Figure 3c) for the development of a whole plant (Figure 3d). Well‐developed shoots and roots were observed in the regenerated plants after growing on RI media for 4 weeks (Figure 3e,f). The well‐developed plants were acclimatized into soil in a controlled growth chamber (Figure 3g), and further grown in a greenhouse to produce T1 seeds (Figure 3h). For transformation with pKSE401‐sgRNA1, 34 kanamycin‐resistant plants were regenerated from 176 explants with an efficiency of 19.3%. For transformation with pKSE401‐sgRNA1+2, 48 kanamycin‐resistant plants were regenerated from 238 explants with an efficiency of 20.2%. Twenty‐four plants transformed with pKSE401‐sgRNA1 and 32 plants transformed with pKSE401‐sgRNA1+2 were tested for the integration of the transgene by PCR. All transformed plants were shown to be positive for the integration of their respective transgene (Figure S2).

### 3.4 Targeted mutagenesis of ObDMR1 in T0 transgenic plants

**FIGURE 4:**
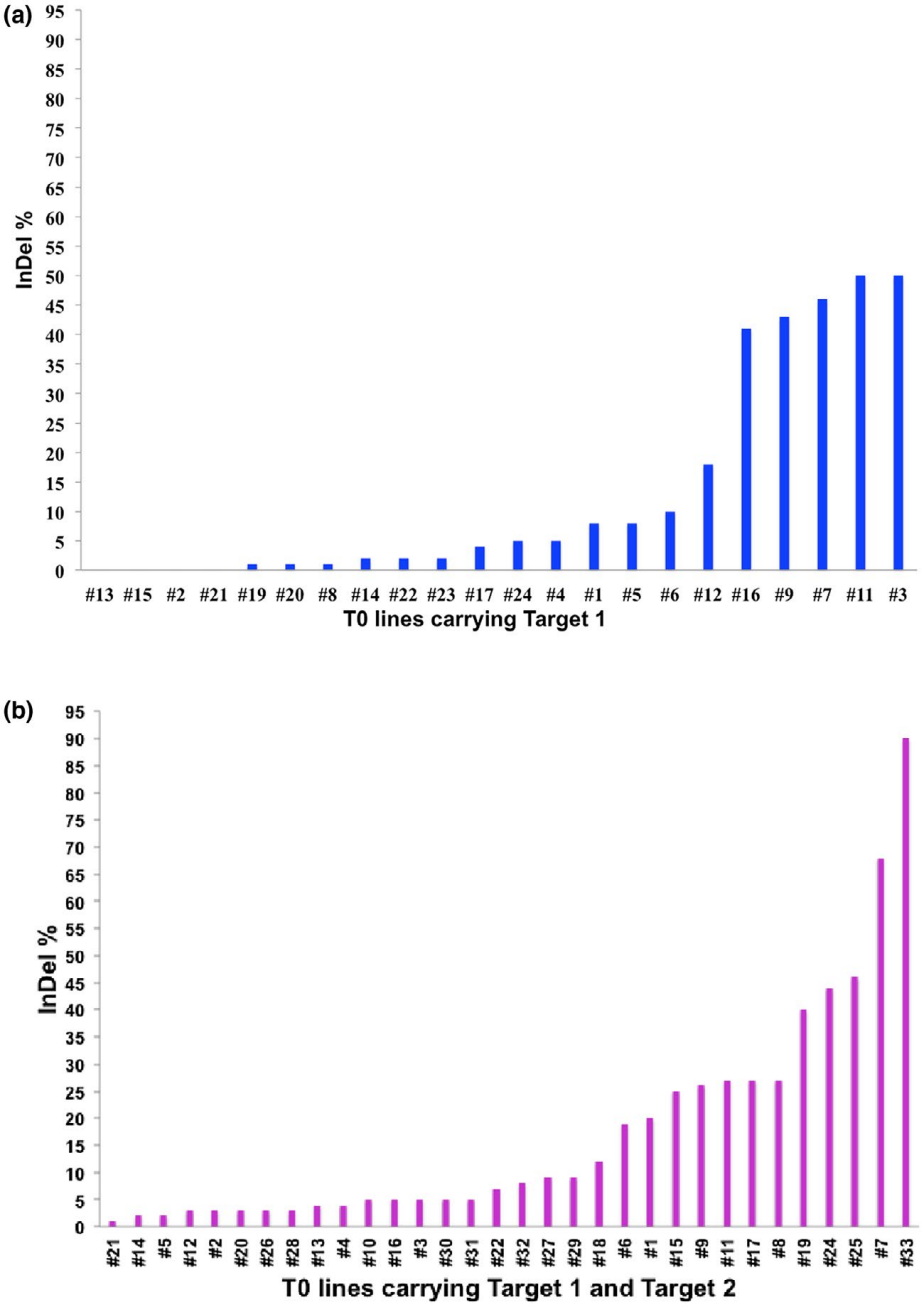
Indel frequency (%) at target site 1 in T0 transgenic lines transformed with the CRISPR/Cas9 constructs expressing one sgRNA (a) or two sgRNAs (b). The data were generated by decoding the Sanger sequencing chromatograms of the 383‐bp *ObDMR1* fragments amplified from each line using The Synthego ICE v1.1 CRISPR Analysis Tool (https://ice.synthego.com/). CRISPR, clustered regularly interspaced short palindromic repeat; sgRNA, single guide RNA

To identify the mutations of *ObDMR1* in transgenic plants, a 383‐bp fragment spanning both sgRNA target sites (Figure 2a) were amplified by PCR and subjected to Sanger sequencing. The chromatograms were decoded using Synthego ICE v1.1 CRISPR Analysis Tool (Hsiau et al., 2018) to assess the indel frequency. Among the 24 T0 plants carrying only sgRNA1, we were able to obtain good chromatograms for analysis for 22 lines. No mutation was detected in four lines (#13, #15, #2, #21), while varied percentages of indel mutations were detected in the remaining 18 lines, leading to an 81.8% mutation rate (Figure 4a). A similar analysis was run for the 32 T0 transgenic lines transformed with the construct expressing two sgRNAs. A 100% mutation rate was achieved with different percentages of indels detected at sgRNA1 target site in each individual plant (Figure 4b). As sweet basil is tetraploid (Pyne et al., 2018), T0 plants with indel percentage less than 25% can be attributed as the ones having chimeric mutations. The plants with relatively high indel frequency (≥50%) were selected for further downstream experiments. Of the 10 such lines, four died during the acclimatization period. The remaining six, including #9, #11, and #16 carrying only sgRNA1, and #7, #24, and #33 carrying two sgRNAs, were selected for amplicon deep sequencing.

**FIGURE 5:**
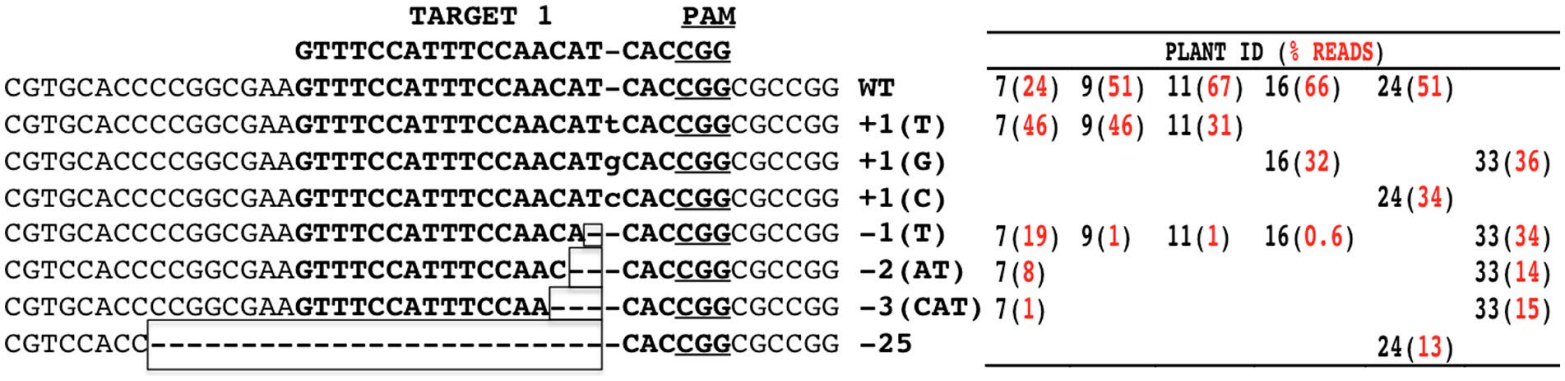
Mutation types at target 1 site and distribution in six independent T0 transgenic lines, determined by amplicon deep sequencing. The different insertions are marked in lower case and deletions are indicated using boxed dashed lines. The 20‐nt target sequence of target 1 and the downstream PAM site (underlined) are shown in bold. The identities (IDs) of T0 transgenic plants are shown in numbers, with 9, 11, and 16 carrying one sgRNA, and 7, 24, and 33 carrying two sgRNAs. The percentage of reads corresponding to a mutation type in each line is shown using a number in red in a parenthesis. sgRNA, single guide RNA

Amplicon deep sequencing of the 383‐bp *ObDMR1* fragments from each of the six lines yielded around 50,000 high‐quality reads. A significant percentage of reads contained mutations in sgRNA1 target site (Figure 5). Although mutations were also detected in the sgRNA2 target site for the three lines that carry two sgRNAs, the percentage of reads with such mutations was lower than 0.5%. The prevalent editions at sgRNA1 target site included short insertions (+1 bp) and deletions (−1, −2, or −3 bp) as well as a larger deletion (−25 bp). The 1‐bp insertion was the most common mutation type and appeared in all six lines that were subjected to amplicon sequencing, while 1–3‐bp deletions were detected in five lines except #24 (Figure 5). 13% of the amplicon reads in T0 line #24 contained a 25‐bp deletion at the target 1 site. A significant percentage of reads contained no mutations in five lines except #33. Line #33 contained four major types of mutations and a negligible amount (0.01%) of WT reads, which suggests that it is a complete knockout mutant (Figure 5). This indicates that the CRISPR/Cas9‐mediated gene editing system in sweet basil is highly efficient, with the capacity to generate complete knockout mutants in the first generation of transgenic plants.

### 3.5 Obtaining of transgene‐free homozygous mutants in T1 generation

**FIGURE 6:**
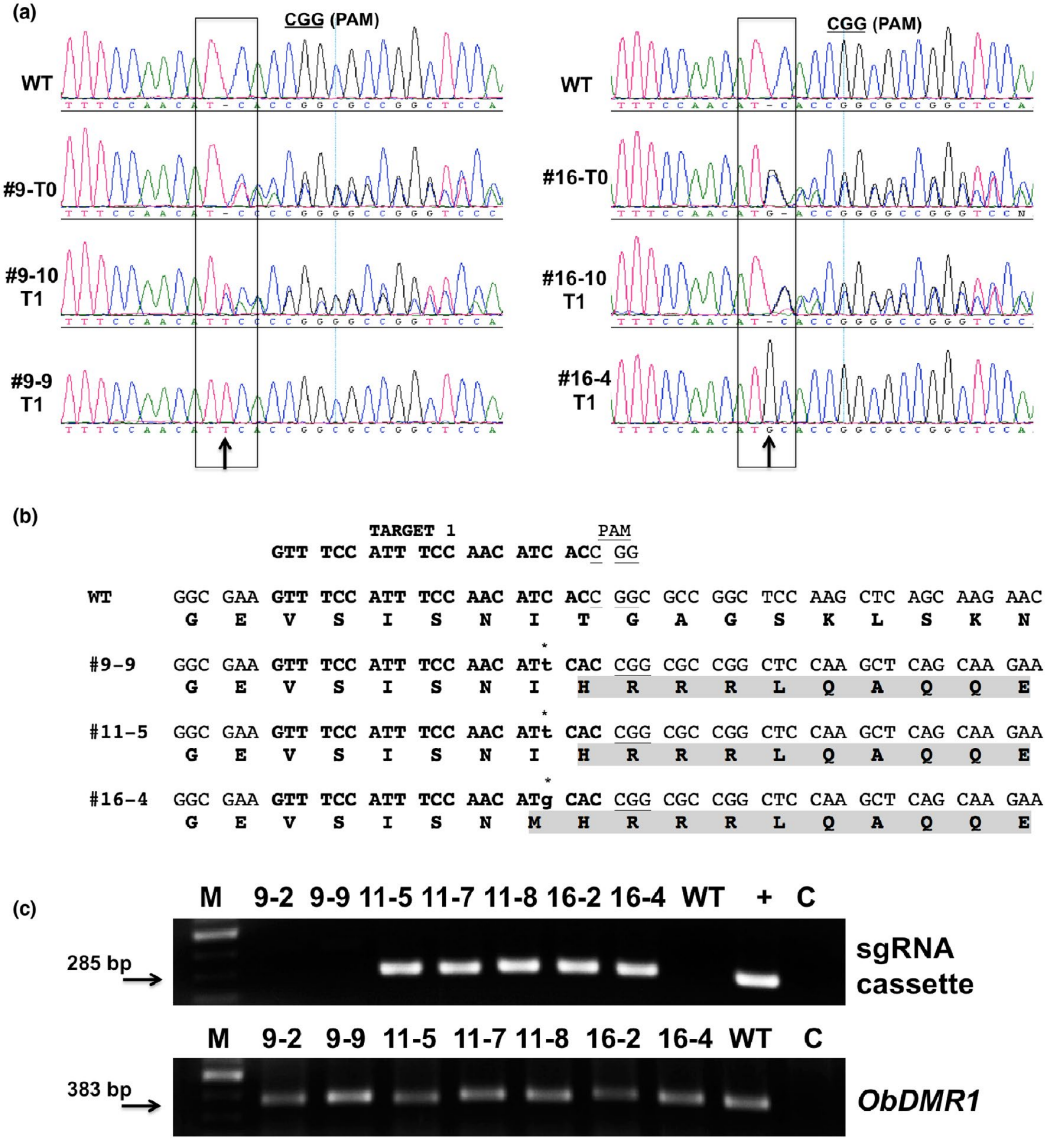
Characterization of *ObDMR1* homozygous and/or transgene‐free mutants in T1 generation. (a) Chromatograms of the *ObDMR1* fragments from the wild‐type (WT), T0 plants #9 and #16 (#9‐T0, #16‐T0) and their derived T1 plants with heterozygous (#9‐10, #16‐10) and homozygous mutations (#9‐9 and #16‐4). The inserted “T” and “G” are indicated with an arrow. (b) Nucleotide insertions at target 1 and the resultant amino‐acid changes in T1 homozygous mutants indicated. The 20‐nt sgRNA1 target sequence is shown in bold with PAM underlined. The inserted nucleotides are shown in lower case below asterisks, the mutated amino acids are shaded. (c) Agarose gel images showing PCR amplification of the sgRNA cassette (upper panel) and *ObDMR1* (lower panel) from WT and indicated homozygous T1 lines. +, pKSE401‐sgRNA1 as a template; C, no template negative control; M, 100bp ladder. The sizes of the corresponding bands are indicated with arrows. sgRNA, single guide RNA

The three T0 lines (#9, #11, and #16) that contained only sgRNA1 were self‐fertilized to produce seeds of T1 generation. *ObDMR1* mutations in T1 plants were detected by sequencing the 383‐bp *ObDMR1* fragments through Sanger sequencing. The chromatograms obtained from individual T1 plants revealed that the mutations in T0 plants were inherited and segregated. T1 plants showing chromatogram with overlapping peaks same as T0 starting from the Cas9 cleavage site (3‐nt upstream of PAM) were considered as heterozygous for the mutation whereas T1 plants with clear insertion of a new nucleotide close to the Cas9 cleavage site were regarded as homozygous mutants (Figure 6a). For each T0 line, we tested 10–12 T1 plants for *ObDMR1* mutations. We detected 2–3 T1 homozygous mutants derived from each T0 line. In total, seven T1 homozygous mutant plants were obtained. T1 homozygous mutants derived from T0 lines #9 (#9‐2 and #9‐9) and #11 (#11‐5, #11‐7, and #11‐8) contained the same mutation with a “T” inserted immediately before the Cas9 cleavage site, while T1 homozygous mutants derived from T0 line #16 (#16‐2 and #16‐4) contained a “G” insertion (Figure 6a). These mutations resulted in *ObDMR1* ORF shift and correspondingly altered the amino acid sequence (Figure 6b).

To identify transgene‐free homozygous mutants, we determined the presence of transgene in the seven T1 homozygous mutants by amplifying a fragment of sgRNA1 expression cassette delivered by the plasmid pKSE401‐sgRNA1 (Figure 2b). As a template integrity control, the 383‐bp *ObDMR1* fragment was successfully amplified from all plants (Figure 6c). No amplification of the sgRNA1 expression cassette was observed in T1 line #9‐2 and #9‐9, whereas its amplification in other lines was successful (Figure 6c), demonstrating that the transgene was segregated out from #9‐2 and #9‐9. These results demonstrated the heritability of *ObDMR1* mutations to next generation independent of the presence of transgene, which leads to transgene‐free homozygous mutants.

### 3.6 Dwarf phenotype of homozygous ObDMR1 mutants at seedling stage

**FIGURE 7:**
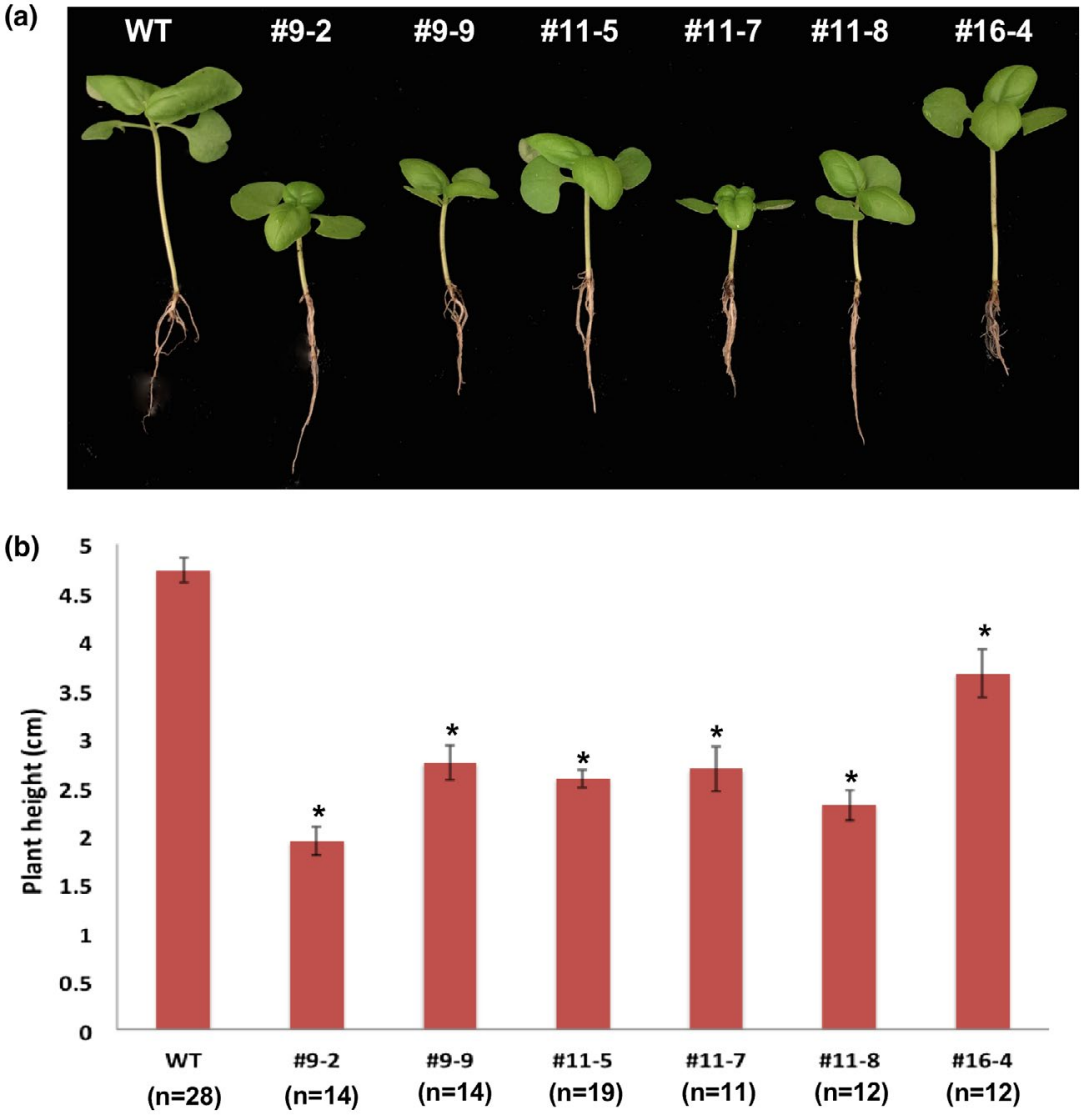
Stunted growth of *ObDMR1* homozygous mutants at young seedling stage. (a) Photographs of a representative T2 plant derived from each individual T1 homozygous mutant line indicated and the wild‐type (WT). (b) Average plant height of T2 homozygous mutants and WT. 18‐day‐old plants were used to take photographs and measurements. The number of plants measured for each line is indicated in parenthesis. Error bars represent standard error of the mean. The asterisks (*) indicate statistically significant differences (*p* < .001) between the mutant line and WT, determined by one‐tailed *t* test. #9‐2, #9‐9, #11‐5, #11‐7 and #11‐8 contain 1‐bp “T” insertion and #16‐4 carries 1‐bp “G” insertion in *ObDMR1*. Similar stunted growth was consistently observed when T2 seeds of these mutant lines were sown and grown side by side with WT

In order to observe the morphological phenotypic differences under normal growth conditions, T2 homozygous *ObDMR1* mutants along with the WT plants were sown and grown in the same controlled growth chambers. Homozygous mutant seeds germinated normally and no clear morphological difference between the mutants and WT was observed within 2 weeks after sowing. However, varying levels of stunted growth started to appear in seedlings of *ObDMR1* mutants after that. At 18 days old, all mutant lines displayed apparent dwarfism compared with WT, with 9–2, 9–9, 11–5, 11–7, and 11–8 more drastic than 16–4 (Figure 7). The notable stunting in plant height was observed at young seedling stage. The difference in height gradually reduced along with age. Mutant plants of 45 days old or older showed similar growth and expansion of leaves as WT.

## 4 DISCUSSION

Clustered regularly interspaced short palindromic repeat‐mediated genome editing in plants not only provides a powerful tool for plant functional genomics studies, but also effectively enhances plant breeding (Arora & Narula, 2017; Jaganathan et al., 2018). As sweet basil is an economically important herb that produces essential oil containing many high‐value secondary metabolites, functional understanding of the synthetic pathways of these metabolites is very important for improving their production and application. Currently, production of sweet basil is largely hampered due to the downy mildew disease caused by the obligate biotrophic oomycete, *P. belbahrii*. Developing highly effective and environmentally friendly disease management strategies requires the fundamental understanding of basil‐*P. belbahrii* molecular interactions. As such, effective functional genomics tools for sweet basil are in dire need. In this study, we have established a CRISPR/Cas9‐mediated gene editing system to effectively generate homozygous and/or complete knockout sweet basil mutants. This system takes advantage of a binary plasmid pKSE401, which was previously developed for gene editing in dicots (Xing et al., 2014). By expressing maize‐codon optimized Cas9 under the control of the double CaMV 35S promoter and sgRNA1 under AtU6‐26 promoter using *Agrobacterium*‐mediated transformation, we were able to generate mutations at target 1 site in 50 out of 54 tested T0 transgenic sweet basil plants, with a 92.6% mutation rate (Figure 4). Amplicon deep sequencing of six selected T0 plants revealed diverse types of indels produced and identified one complete knockout mutant. This reflects the high efficiency of the CRISPR/Cas9 system we established for sweet basil, having the capacity to create complete knockout mutants of this tetraploid species at the first generation of transgenic plants. All procedures from target design to identification of mutant T0 plants can be achieved in 4–5 months (Figure S3). In addition, we were able to readily identify homozygous mutants in the second generation (T1) of T0 transgenic plants with heterozygous and/or chimeric mutations. Furthermore, we were able to identify homozygous mutant plants free of the transgene in the T1 generation. Altogether, this study established a highly efficient gene editing system for sweet basil, which is expected to accelerate sweet basil functional genomics and enable breeding of consumer‐acceptable varieties with desirable traits in a short time frame. The system established may be applicable for other *Ocimum* species.

*ObDMR1* homozygous mutant plants derived from three independent T0 lines displayed stunted growth at young seedling stage. This is consistent with previous observations in other plant species when *DMR1* homologs were mutated or silenced. Reduced growth was previously observed in *Arabidopsis* recessive homozygous *dmr1* mutants (Van Damme et al., 2005, 2009). Both transgenic tomato and potato plants with *DMR1* homologs silenced exhibited dwarfing stature (Huibers et al., 2013; Sun et al., 2016). The dwarfing phenotypes of these *ObDMR1* mutant plants further confirmed that complete knockout of *ObDMR1* was successfully achieved using CRISPR/Cas9‐mediated gene editing. *ObDMR1* is probably implicated in the regulation of plant height during the seedling stage similar to its ortholog in *Arabidopsis* (Van Damme et al., 2005). Although the height of all tested homozygous mutant lines was significantly shorter than WT, T1 homozygous lines #9‐2, #9‐9, #11‐5, #11‐7, and #11‐8 that carry a “T” insertion displayed greater dwarfing phenotypic aberration than #16‐4 that carries a “G” insertion (Figure 7). It is unclear what caused the difference. The “T” and “G” insertion are located at the same location of the gene and the resultant frameshifts lead to the change to the same amino acid sequence except for the codon containing the nucleotide insertion (Figure 6b). Insertion of “T” did not change the amino acid isoleucine (I), while insertion of “G” led to the change from I to methionine (M) (Figure 6b). This single amino acid difference may somehow relieve the dwarfing effect. Another possibility is that the integration of the transgene in #16‐4 disrupted a gene that plays a role in regulating plant height. The second possibility can be tested using a transgene‐free line with “G” homozygous insertion, which is currently being identified from a T1 population of T0 line #16. While the dwarfing phenotype of *ObDMR1* mutants analyzed in this study is similar to that seen in plants knocked out or silenced for *DMR1* in other species, this needs to be further confirmed using transgene‐free plants derived from multiple independent transgenic T0 lines. Our assay included transgene‐free homozygous mutant lines #9‐2 and #9‐9 that were derived from a single T0 line. Using transgene‐free plants derived from additional T0 lines and meanwhile analyzing the association of the phenotype with *ObDMR1* mutation in segregating progenies are necessary for a firm validation.

Although mutations were effectively generated at sgRNA1 target site, very low to negligible frequency of mutations occurred at target site 2. It is unclear what caused the significant difference. The target sequence could be one contributing factor. Previous studies showed that CRISPR/Cas9‐induced mutation efficiency in plants is affected by the GC content and secondary structure of sgRNAs (Ma et al., 2015; Tang et al., 2018). In our study, we used two 20‐nt sgRNA target sequences with varying GC content of 40% and 55%, respectively, and different secondary structures (Table S1). Another factor could be due to the effectiveness of the promoter that drives the expression of sgRNAs. A report demonstrated that AtU6‐26 promoter displayed a much higher transcriptional activity in model plant *Arabidopsis* than AtU6‐29 promoter (Li, Jiang, Yong, & Zhang, 2007). In our study AtU6‐26 and AtU6‐29 were used to drive the expression of sgRNA1 and sgRNA2, respectively. The low expression of sgRNA2 may have led to the low efficiency of mutations at target 2. Determining the expression levels of both sgRNA1 and sgRNA2 using reverse transcription quantitative PCR (RT‐qPCR) will clarify this and determine whether AtU6‐29 promoter is suitable to be used for multiplexing gene editing in sweet basil. At this point, since AtU6‐26 promoter was shown to be highly effective in sweet basil gene editing, multiplexing gene editing can be achieved by expressing an array of sgRNAs interspaced with tRNA as a single polycistronic gene under AtU6‐26 promoter, which is then processed to multiple sgRNAs through the endogenous tRNA processing system (Xie, Minkenberg, & Yang, 2015). Alternatively, multiplex gene editing may be achieved using CRISPR/Cas12 system as the dual nuclease activity of Cas12 allows the processing of a single transcript containing multiple guide RNAs and simultaneous editing of their targeted loci (Wang, Mao, Lu, Tao, & Zhu, 2017).

The whole genome sequence assembly for sweet basil has been generated, but not yet publicly available (Dudai et al., 2018). As such, we were not able to analyze the off‐targets in detail. We tried to limit the off‐targets based on the transcriptomic sequences by selecting the target sequences without the off‐target in the transcriptome through sgRNA design tool EuPaGDT (Peng & Tarleton, 2015). However, detailed analysis of off‐targets need to be done in the future when the whole genome sequence of sweet basil is available.

Mutation or silencing of *DMR1* homologs in *Arabidopsis*, tomato, and potato confers resistance to *Arabidopsis* downy mildew *H. arabidopsidis* (Van Damme et al., 2005, 2009), tomato powdery mildew pathogen *Oidium neolycopersici* (Huibers et al., 2013), and potato late blight pathogen *Phytophthora infestans* (Sun et al., 2016), respectively. Infection assays are currently underway to determine whether *ObDMR1* homozygous mutants also confer resistance to BDM pathogen *P. belbahrii*.

## CONFLICT OF INTEREST

The authors declare no conflict of interest to this work.

## AUTHOR CONTRIBUTIONS

M. Tian conceived and designed the overall study. N. Navet designed and performed the experiments, and analyzed the data. N. Navet wrote the initial draft of the manuscript. M. Tian reviewed and edited the manuscript. Both authors approved the final manuscript.

## Supporting information

Figs S1‐S3‐Table S1

## References

[pld3233-bib-0001] Arora, L. , & Narula, A. (2017). Gene editing and crop improvement using CRISPR‐Cas9 system. Frontiers in Plant Science, 8, 1932 10.3389/fpls.2017.01932 29167680 PMC5682324

[pld3233-bib-0002] Ben‐Naim, Y. , Falach, L. , & Cohen, Y. (2018). Transfer of downy mildew resistance from wild basil (*Ocimum americanum*) to sweet basil (*O. basilicum*). Phytopathology, 108, 114–123. 29083273 10.1094/PHYTO-06-17-0207-R

[pld3233-bib-0003] Borrelli, V. M. G. , Brambilla, V. , Rogowsky, P. , Marocco, A. , & Lanubile, A. (2018). The enhancement of plant disease resistance using CRISPR/Cas9 technology. Frontiers in Plant Science, 9, 1245 10.3389/fpls.2018.01245 30197654 PMC6117396

[pld3233-bib-0004] Cohen, Y. , Ben Naim, Y. , Falach, L. , & Rubin, A. E. (2017). Epidemiology of basil Downy Mildew. Phytopathology, 107, 1149–1160. 10.1094/PHYTO-01-17-0017-FI 28437138

[pld3233-bib-0005] da Costa, A. S. , Arrigoni‐Blank, M. F. , de Carvalho Filho, J. L. S. , de Santana, A. D. D. , de Alexandria Santos, D. , Alves, P. B. , & Blank, A. F. (2015). Chemical diversity in basil germplasm. The Scientific World Journal, 2015, 352638. 25629084 10.1155/2015/352638 PMC4299303

[pld3233-bib-0006] Deschamps, C. , & Simon, J. E. (2002). *Agrobacterium tumefaciens*‐mediated transformation of *Ocimum basilicum* and *O. citriodorum* . Plant Cell Reports, 21, 359–364. 10.1007/s00299-002-0526-0

[pld3233-bib-0007] Dong, O. X. , & Ronald, P. C. (2019). Genetic engineering for disease resistance in plants: Recent progress and future perspectives. Plant Physiology, 180, 26–38. 10.1104/pp.18.01224 30867331 PMC6501101

[pld3233-bib-0008] Dudai, N. , Carp, M. J. , Milavski, R. , Chaimovitsh, D. , Shachter, A. , Baruch, K. , … Gonda, I. (2018). High‐quality assembly of sweet basil genome. bioRxiv. 10.1101/476044

[pld3233-bib-0009] Gumtow, R. , Wu, D. , Uchida, J. , & Tian, M. (2018). A *Phytophthora palmivora* extracellular cystatin‐like protease inhibitor targets papain to contribute to virulence on papaya. Molecular Plant‐Microbe Interactions, 31, 363–373. 29068239 10.1094/MPMI-06-17-0131-FI

[pld3233-bib-0010] Haque, E. , Taniguchi, H. , Hassan, M. M. , Bhowmik, P. , Karim, M. R. , Smiech, M. , … Islam, T. (2018). Application of CRISPR/Cas9 genome editing technology for the improvement of crops cultivated in tropical climates: Recent progress, prospects, and challenges. Frontiers in Plant Science, 9, 617 10.3389/fpls.2018.00617 29868073 PMC5952327

[pld3233-bib-0011] Hok, S. , Danchin, E. G. , Allasia, V. , Panabieres, F. , Attard, A. , & Keller, H. (2011). An *Arabidopsis* (malectin‐like) leucine‐rich repeat receptor‐like kinase contributes to downy mildew disease. Plant, Cell and Environment, 34, 1944–1957. 10.1111/j.1365-3040.2011.02390.x 21711359

[pld3233-bib-0012] Hsiau, T. , Maures, T. , Waite, K. , Yang, J. , Kelso, R. , Holden, K. , & Stoner, R. (2018). Inference of CRISPR edits from Sanger trace data. bioRxiv. 10.1101/251082 35119294

[pld3233-bib-0013] Huibers, R. P. , Loonen, A. E. , Gao, D. , Van den Ackerveken, G. , Visser, R. G. , & Bai, Y. (2013). Powdery mildew resistance in tomato by impairment of *SlPMR4* and *SlDMR1* . PLoS One, 8, e67467 10.1371/journal.pone.0067467 23818978 PMC3688610

[pld3233-bib-0014] Jaganathan, D. , Ramasamy, K. , Sellamuthu, G. , Jayabalan, S. , & Venkataraman, G. (2018). CRISPR for crop improvement: An update review. Frontiers in Plant Science, 9, 985 10.3389/fpls.2018.00985 30065734 PMC6056666

[pld3233-bib-0015] Jiang, F. , & Doudna, J. A. (2017). CRISPR‐Cas9 structures and mechanisms. Annual Review of Biophysics, 46, 505–529. 10.1146/annurev-biophys-062215-010822 28375731

[pld3233-bib-0016] Jinek, M. , Chylinski, K. , Fonfara, I. , Hauer, M. , Doudna, J. A. , & Charpentier, E. (2012). A programmable dual‐RNA‐guided DNA endonuclease in adaptive bacterial immunity. Science, 337, 816–821. 10.1126/science.1225829 22745249 PMC6286148

[pld3233-bib-0017] Jinek, M. , East, A. , Cheng, A. , Lin, S. , Ma, E. , & Doudna, J. (2013). RNA‐programmed genome editing in human cells. Elife, 2, e00471 10.7554/eLife.00471 23386978 PMC3557905

[pld3233-bib-0018] Langner, T. , Kamoun, S. , & Belhaj, K. (2018). CRISPR crops: Plant genome editing toward disease resistance. Annual Review of Phytopathology, 56, 479–512. 10.1146/annurev-phyto-080417-050158 29975607

[pld3233-bib-0019] Larkin, M. A. , Blackshields, G. , Brown, N. P. , Chenna, R. , McGettigan, P. A. , McWilliam, H. , … Higgins, D. G. (2007). Clustal W and Clustal X version 2.0. Bioinformatics, 23, 2947–2948. 10.1093/bioinformatics/btm404 17846036

[pld3233-bib-0020] Li, Q. X. , & Chang, C. L. (2016). Basil (*Ocimum basilicum* L.) oils In Preedy V. R. (Ed.), Essential oils in food preservation, flavor and safety (pp. 231–238). Cambridge, MA: Academic Press.

[pld3233-bib-0021] Li, X. , Jiang, D. H. , Yong, K. , & Zhang, D. B. (2007). Varied trancriptional efficiencies of multiple *Arabidopsis* U6 small nuclear RNA genes. Journal of Integrative Plant Biology, 49, 222–229.

[pld3233-bib-0022] Ma, X. , Zhang, Q. , Zhu, Q. , Liu, W. , Chen, Y. , Qiu, R. , … Liu, Y. G. (2015). A robust CRISPR/Cas9 system for convenient, high‐efficiency multiplex genome editing in monocot and dicot plants. Molecular Plant, 8, 1274–1284. 10.1016/j.molp.2015.04.007 25917172

[pld3233-bib-0023] Marchler‐Bauer, A. , Bo, Y. , Han, L. , He, J. , Lanczycki, C. J. , Lu, S. , … Bryant, S. H. (2017). CDD/SPARCLE: Functional classification of proteins via subfamily domain architectures. Nucleic Acids Research, 45, D200–D203. 10.1093/nar/gkw1129 27899674 PMC5210587

[pld3233-bib-0024] Pan, C. , Ye, L. , Qin, L. , Liu, X. , He, Y. , Wang, J. , … Lu, G. (2016). CRISPR/Cas9‐mediated efficient and heritable targeted mutagenesis in tomato plants in the first and later generations. Scientific Reports, 6, 24765 10.1038/srep24765 27097775 PMC4838866

[pld3233-bib-0025] Peng, D. , & Tarleton, R. (2015). EuPaGDT: A web tool tailored to design CRISPR guide RNAs for eukaryotic pathogens. Microb Genom, 1, e000033 10.1099/mgen.0.000033 28348817 PMC5320623

[pld3233-bib-0026] Phippen, W. B. , & Simon, J. E. (2000). Shoot regeneration of young leaf explants from basil (*Ocimum basilicum* L.). In Vitro Cellular & Developmental Biology ‐ Plant, 36(4), 250–254. 10.1007/s11627-000-0046-y

[pld3233-bib-0027] Pyne, R. M. , Honig, J. A. , Vaiciunas, J. , Wyenandt, C. A. , & Simon, J. E. (2018). Population structure, genetic diversity and downy mildew resistance among *Ocimum* species germplasm. BMC Plant Biology, 18, 69 10.1186/s12870-018-1284-7 29685108 PMC5914031

[pld3233-bib-0028] Reuter, J. S. , & Mathews, D. H. (2010). RNAstructure: software for RNA secondary structure prediction and analysis. BMC Bioinformatics, 11(1), 1471–2105. 10.1186/1471-2105-11-129 PMC2984261 20230624

[pld3233-bib-0029] Shao, D. , & Tian, M. (2018). A qPCR approach to quantify the growth of basil downy mildew pathogen *Peronospora belbarhii* during infection. Current Plant Biology, 15, 2–7.

[pld3233-bib-0030] Sun, K. , Wolters, A. M. , Vossen, J. H. , Rouwet, M. E. , Loonen, A. E. , Jacobsen, E. , … Bai, Y. (2016). Silencing of six susceptibility genes results in potato late blight resistance. Transgenic Research, 25, 731–742. 10.1007/s11248-016-9964-2 27233778 PMC5023794

[pld3233-bib-0031] Tang, T. , Yu, X. , Yang, H. , Gao, Q. , Ji, H. , Wang, Y. , … Dai, C. (2018). Development and validation of an effective CRISPR/Cas9 vector for efficiently isolating positive transformants and transgene‐free mutants in a wide range of plant species. Frontiers in Plant Science, 9, 1533 10.3389/fpls.2018.01533 30405669 PMC6206294

[pld3233-bib-0032] Torre, S. , Tattini, M. , Brunetti, C. , Guidi, L. , Gori, A. , Marzano, C. , … Sebastiani, F. (2016). *De novo* assembly and comparative transcriptome analyses of red and green morphs of sweet basil grown in full sunlight. PLoS One, 11, e0160370 10.1371/journal.pone.0160370 27483170 PMC4970699

[pld3233-bib-0033] Van Damme, M. , Andel, A. , Huibers, R. P. , Panstruga, R. , Weisbeek, P. J. , & Van den Ackerveken, G. (2005). Identification of *Arabidopsis* loci required for susceptibility to the downy mildew pathogen *Hyaloperonospora parasitica* . Molecular Plant‐Microbe Interactions, 18, 583–592. 15986928 10.1094/MPMI-18-0583

[pld3233-bib-0034] van Damme, M. , Huibers, R. P. , Elberse, J. , & Van den Ackerveken, G. (2008). *Arabidopsis* DMR6 encodes a putative 2OG‐Fe(II) oxygenase that is defense‐associated but required for susceptibility to downy mildew. The Plant Journal, 54, 785–793. 10.1111/j.1365-313X.2008.03427.x 18248595

[pld3233-bib-0035] van Damme, M. , Zeilmaker, T. , Elberse, J. , Andel, A. , de Sain‐van der Velden, M. , & van den Ackerveken, G. (2009). Downy mildew resistance in *Arabidopsis* by mutation of HOMOSERINE KINASE. The Plant Cell, 21, 2179–2189. 19622802 10.1105/tpc.109.066811 PMC2729605

[pld3233-bib-0036] van Schie, C. C. , & Takken, F. L. (2014). Susceptibility genes 101: How to be a good host. Annual Review of Phytopathology, 52, 551–581. 10.1146/annurev-phyto-102313-045854 25001453

[pld3233-bib-0037] Waltz, E. (2016). Gene‐edited CRISPR mushroom escapes US regulation. Nature, 532(7599), 293 10.1038/nature.2016.19754 27111611

[pld3233-bib-0038] Wang, F. , Wang, C. , Liu, P. , Lei, C. , Hao, W. , Gao, Y. , … Zhao, K. (2016). Enhanced rice blast resistance by CRISPR/Cas9‐targeted mutagenesis of the ERF transcription factor gene OsERF922. PLoS One, 11, e0154027 10.1371/journal.pone.0154027 27116122 PMC4846023

[pld3233-bib-0039] Wang, M. , Mao, Y. , Lu, Y. , Tao, X. , & Zhu, J. K. (2017). Multiplex gene editing in rice using the CRISPR‐Cpf1 System. Molecular Plant, 10, 1011–1013. 10.1016/j.molp.2017.03.001 28315752

[pld3233-bib-0040] Wyenandt, C. A. , Simon, J. E. , Pyne, R. M. , Homa, K. , McGrath, M. T. , Zhang, S. , … Madeiras, A. (2015). Basil downy mildew (*Peronospora belbahrii)*: Discoveries and challenges relative to its control. Phytopathology, 105, 885–894. 25894318 10.1094/PHYTO-02-15-0032-FI

[pld3233-bib-0041] Xie, K. , Minkenberg, B. , & Yang, Y. (2015). Boosting CRISPR/Cas9 multiplex editing capability with the endogenous tRNA‐processing system. Proceedings of the National Academy of Sciences of the United States of America, 112, 3570–3575. 10.1073/pnas.1420294112 25733849 PMC4371917

[pld3233-bib-0042] Xing, H. L. , Dong, L. , Wang, Z. P. , Zhang, H. Y. , Han, C. Y. , Liu, B. , … Chen, Q. J. (2014). A CRISPR/Cas9 toolkit for multiplex genome editing in plants. BMC Plant Biology, 14, 327 10.1186/s12870-014-0327-y 25432517 PMC4262988

[pld3233-bib-0043] Zaidi, S. S. , Mukhtar, M. S. , & Mansoor, S. (2018). Genome editing: Targeting susceptibility genes for plant disease resistance. Trends in Biotechnology, 36, 898–906. 10.1016/j.tibtech.2018.04.005 29752192

[pld3233-bib-0044] Zhou, J. , Wang, G. , & Liu, Z. (2018). Efficient genome editing of wild strawberry genes, vector development and validation. Plant Biotechnology Journal, 16, 1868–1877. 10.1111/pbi.12922 29577545 PMC6181217

