# Supplementary material for: Efficient targeted mutagenesis in allotetraploid sweet basil by CRISPR/Cas9
Source: Plant Direct. 2020 Jun 11;4(6):e00233. doi: 10.1002/pld3.233 (PMC7287412; doi:10.1002/pld3.233)
Supplement: Supplementary file 1 — Figs S1‐S3‐Table S1 [file PLD3-4-e00233-s001.pdf]

```
gDNA Genoveser   ATGGCCGCCGTCTGCCTGAAGCTCAACTTCGCCGCCGCCGCCGCCTCCGCCTCCGCAACCACCGTCGCCAACCTATCATCACCAAAGCCCCAAACCC
cDNA RR and T    ------A---------------------------------------------------------------------------------------------
Protein            M  A  A  V  C  L  K  L  N  F  A  A  A  A  A  A  S  A  S  A  T  T  V  A  N  L  S  S  P  K  P  Q  T
                         T

gDNA Genoveser   ACTTAAGATTCAACCCATCCGCATCCGCACTATCAACATCCGCTTATTCCAAATCCACTGAGCCTCTACCCGTCTTCTCCTCCGTCAAATCTTTCGCCCC
cDNA RR and T    ----------------------------------------------------------------------------------------------------
Protein           H  L  R  F  N  P  S  A  S  A  L  S  T  S  A  Y  S  K  S  T  E  P  L  P  V  F  S  S  V  K  S  F  A

gDNA Genoveser   CGCCACCGTCGCCAACTTGGGCCCTGGCTTCGACTTTCTGGGATGCGCCGTAGACGGAATCGGCGACTACGTCAGCCTCCGAGTCGATCCAGACGTGCAC
cDNA RR and T    ------------------------A--------------------------T------------------------------------------------
Protein          P  A  T  V  A  N  L  G  P  G  F  D  F  L  G  C  A  V  D  G  I  G  D  Y  V  S  L  R  V  D  P  D  V  H

gDNA Genoveser   CCCGGCGAAGTTTCCATTTCCAACATCACCGGCGCCGGCTCCAAGCTCAGCAAGAACCCCCTTTGGAATTGCGCCGGCATCGCCGCCATCGCCGTCATGA
cDNA RR and T    --------------------------------------------------------------------C-------------------------------
Protein            P  G  E  V  S  I  S  N  I  T  G  A  G  S  K  L  S  K  N  P  L  W  N  C  A  G  I  A  A  I  A  V  M

gDNA Genoveser   AAATGCTCAGCATCCGCTCCGTGGGTCTCTCGCTCTCTCTCGAAAAGGGCCTCCCTCTGGGCAGCGGCCTCGGCTCCAGCGCGGCCAGCGCCGCCGCAGC
cDNA RR and T    -------------------------------------------------------C-----------------------------T--------------
Protein           K  M  L  S  I  R  S  V  G  L  S  L  S  L  E  K  G  L  P  L  G  S  G  L  G  S  S  A  A  S  A  A  A

gDNA Genoveser   TGCTGTCGCTGTAAACGAGTTGTTCGGGGGTCCTCTGTCGCCGTCAGAACTCGTGTTCGCCGGTCTGGAGTCTGAGGCGAAGGTCTCCGGCTACCACGCG
cDNA RR and T    ---C-----------------------------------------------------------------------------------------------C
Protein          A  A  V  A  V  N  E  L  F  G  G  P  L  S  P  S  E  L  V  F  A  G  L  E  S  E  A  K  V  S  G  Y  H  A

gDNA Genoveser   GACAACGTGGCGCCGTCGATCTTGGGAGGTTTCGTTTTGATACGCAGCTACGACCCTTTGGAACTGATGCAACTAAAGTTTCCCCATGAGAAAAGCTTGT
cDNA RR and T    ----------------------------------------------------------------------------------------------------
Protein            D  N  V  A  P  S  I  L  G  G  F  V  L  I  R  S  Y  D  P  L  E  L  M  Q  L  K  F  P  H  E  K  S  L

gDNA Genoveser   ATTTCGTGCTGGTGAATCCGGAATTCGAAGCCCCAACGAAGAAGATGAGAGCGGCGTTGCCGCAGGAAATCACGATGTCGCACCACATATGGAATTCCAG
cDNA RR and T    ----------------------------------------------------------------------------------------------------
Protein           Y  F  V  L  V  N  P  E  F  E  A  P  T  K  K  M  R  A  A  L  P  Q  E  I  T  M  S  H  H  I  W  N  S

gDNA Genoveser   CCAAGCTGGGGCTTTGGTTGCGTCTGTTTTGCAAGGCGATCTCGTTGGGTTAGGAAAGGCGCTGTCATCGGATAAGATTGTGGAGCCGAAGAGGGCTCCT
mRNA RR and T    ----------------------------------------------------------------------------------------------------
Protein          S  Q  A  G  A  L  V  A  S  V  L  Q  G  D  L  V  G  L  G  K  A  L  S  S  D  K  I  V  E  P  K  R  A  P

gDNA Genoveser   TTGATTCCGGGCATGGAAGCTGTGAAGAAAGCTGCCATCGCAGCAGGGGCGTTTGGTTGCACGATAAGTGGAGCTGGACCAACTGCGGTGGCGGTGACAG
cDNA RR and T    --------------------------------------T--G----------------------------------------------------------
Protein            L  I  P  G  M  E  A  V  K  K  A  A  I  A  A  G  A  F  G  C  T  I  S  G  A  G  P  T  A  V  A  V  T

gDNA Genoveser   ACAGTGAGGAAAAAGGTAGAGAAATTGGGGAGAAAATGGTGGAGGCTTTTGAGAAAGAAGGGAACTTGAAGGCTTTGGCAATGGTGAGGCAGCTTGATAG
cDNA RR and T    ----------------------------------------------------------------------------------------------------
Protein           D  S  E  E  K  G  R  E  I  G  E  K  M  V  E  A  F  E  K  E  G  N  L  K  A  L  A  M  V  R  Q  L  D

gDNA Genoveser   AGTTGGAGCTAGGCTTGTCAGCAGTGTTCCCAGATGA
cDNA RR and T    -------------------------------------
Protein          R  V  G  A  R  L  V  S  S  V  P  R
```

Supplemental Figure 1. Nucleotide and amino acid sequence alignment of *ObDMR1* gDNA from Genoveser and cDNA from Red Rubin (RR) and Tigullio (T). Dashed lines represent identical nucleotides. The amino acid difference was highlighted in yellow.

A. Transgene integration of T0 plants carrying target 1

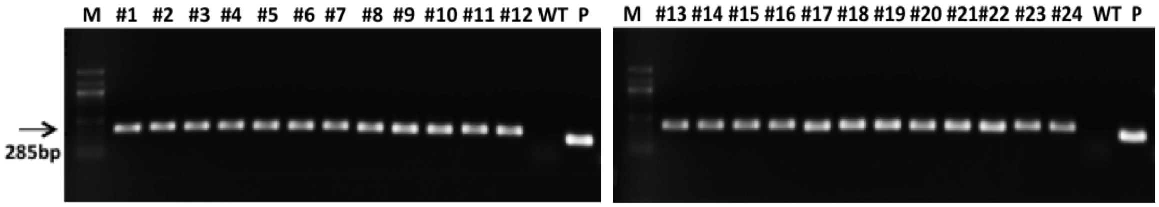

B. Transgene integration of T0 plants carrying target 1 and target 2

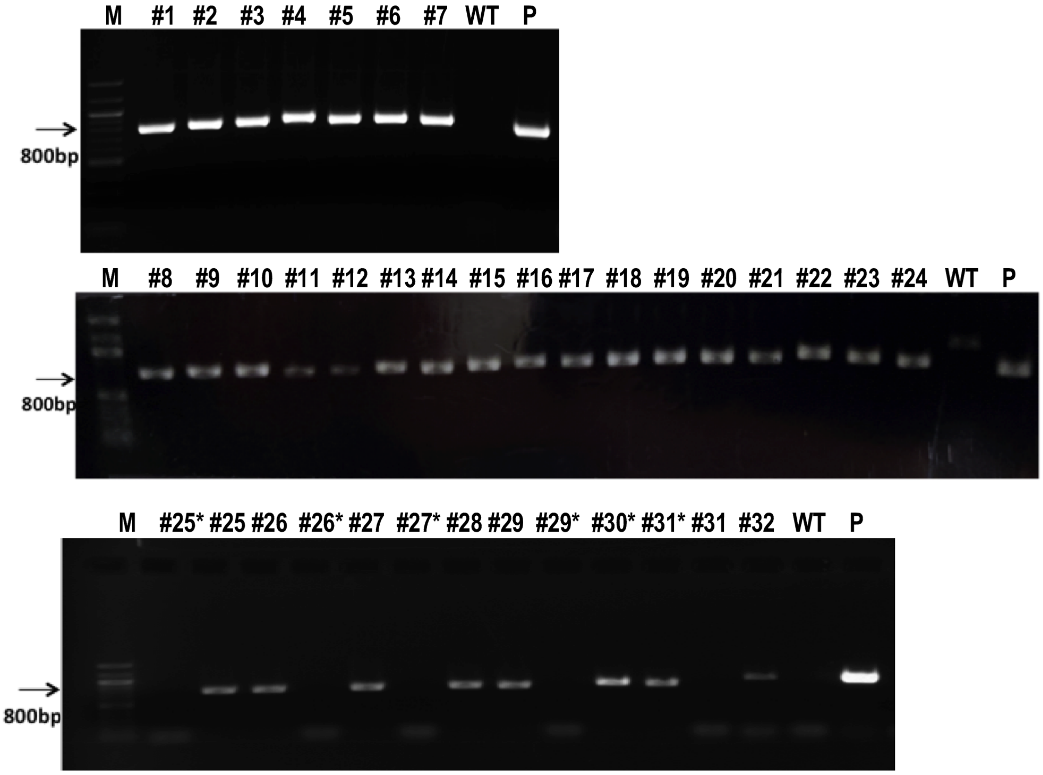

Supplemental Figure 2. Confirmation of T-DNA integration in 56 T0 transgenic plants. ‘M’ indicates 100 bp DNA ladder (NEB), ‘P’ indicates positive controls with pKSE401-sgRNA1 or pKSE401-sgRNA1+2 as a template for T0 plants carrying one sgRNA (top panel) or two sgRNAs (bottom panel). Amplification from the wild-type (WT) plant serves as a negative control. For some lines (the same line number with or without an asterick), two different amounts of gDNA were used for the amplification.

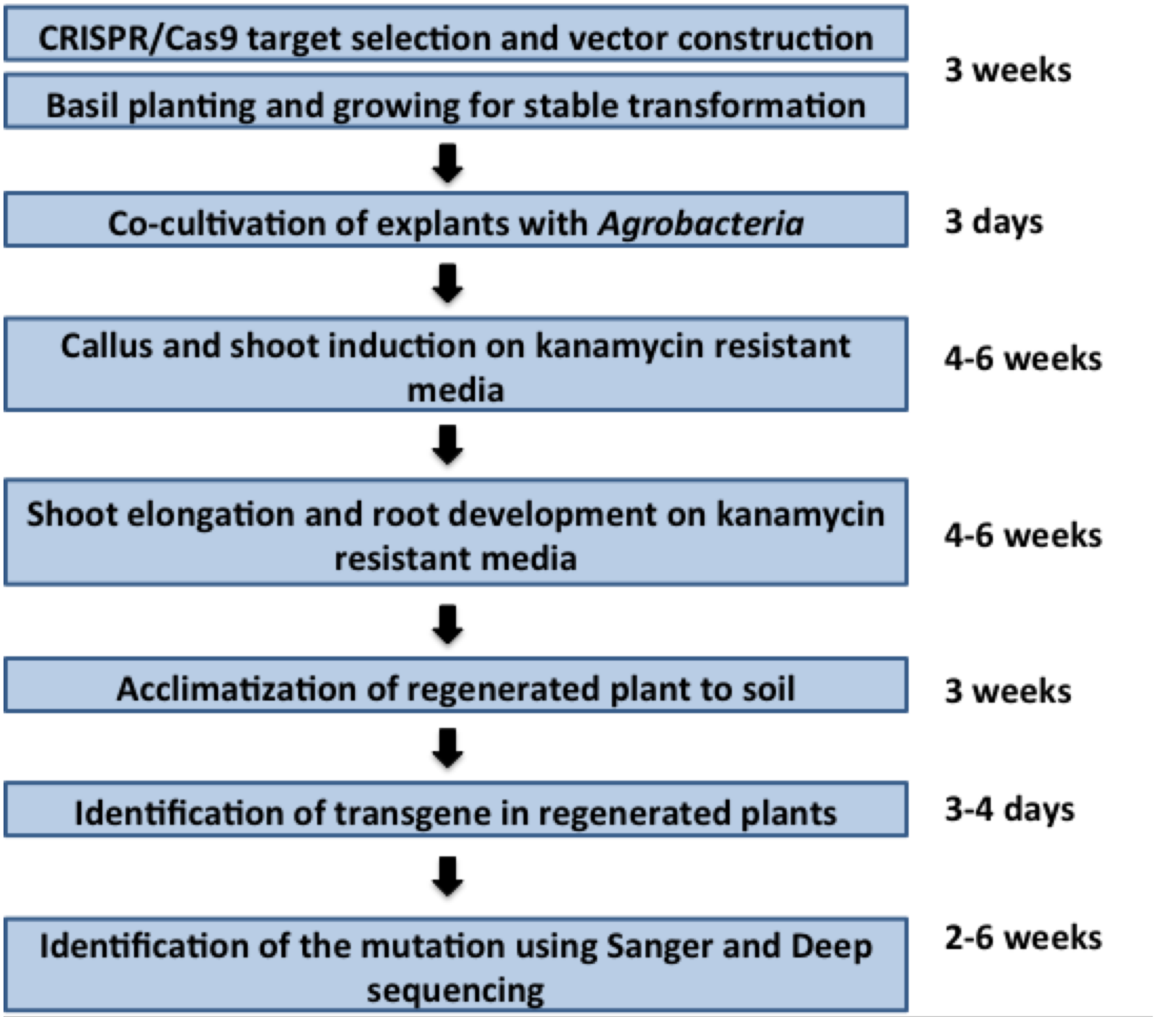

Supplemental Figure 3. A flow chart depicting the major steps and the required time for CRISPR/Cas9-mediated gene editing in sweet basil using *Agrobacterium*-mediated transformation.

Supplemental Table 1. Features of the two selected sgRNA target sequences used for targeted mutagenesis of *ObDMR1*.

TARGET	SEQUENCE	PAM	GC %	TOTAL SCORE	EFFICIENCY SCORE	OFF-TARGET	RNA SECONDARY STRUCTURE
1	GTTTCCATTTCCAACATCAC	CGG	40	0.56	0.55	NO	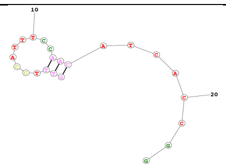
2	GATGCCGGCGCAATTCCAAA	GGG	55	0.54	0.61	NO	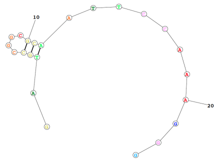
